# Mutations of *MSH5* in nonobstructive azoospermia (NOA) and rescued via in vivo gene editing

**DOI:** 10.1038/s41392-021-00710-4

**Published:** 2022-01-03

**Authors:** Min Chen, Chencheng Yao, Yingying Qin, Xiuhong Cui, Peng Li, Zhiyong Ji, Limei Lin, Haowei Wu, Zhi Zhou, Yaoting Gui, Zheng Li, Fei Gao

**Affiliations:** 1 Guangdong and Shenzhen Key Laboratory of Male Reproductive Medicine and Genetics, Institute of Urology, Peking University Shenzhen Hospital, Shenzhen Peking University-The Hong Kong University of Science and Technology Medical Center, Shenzhen, Guangdong Province P. R. China; 2 Department of Andrology, the Center for Men’s Health, Urologic Medical Center, Shanghai Key Laboratory of Reproductive Medicine, Shanghai General Hospital, Shanghai Jiao Tong University School of Medicine, Shanghai, P. R. China; 3 Center for Reproductive Medicine of Shandong University, National Research Center for Assisted Reproductive Technology and Reproductive Genetics, The Key Laboratory for Reproductive Endocrinology of Ministry of Education, Jinan, P. R. China; 4 State Key Laboratory of Stem cell and Reproductive Biology, Institute of Zoology, Chinese Academy of Sciences, Beijing, P. R. China; 5 University of Chinese Academy of Sciences, Beijing, P. R. China; 6 Institute for Stem Cell and Regeneration, Chinese Academy of Sciences, Beijing, China; 7 School of Life Science and Technology, ShanghaiTech University, Shanghai, P. R. China

**Keywords:** Reproductive disorders, Genetics

**Dear Editor**,

Male infertility is a multifactorial heterogeneous pathological condition affecting ∼7% of men. Nonobstructive azoospermia (NOA) is one of the most severe male reproductive diseases and occurs in ∼1% of men of reproductive age.^1^ However, its etiology remains elusive.

Meiosis is a germ cell-specific cell division process. *MSH5* (MutS homolog 5) is a member of the MutS family and is known to be involved in DNA mismatch repair. MSH5 acts with the MutS homolog partner MSH4 to form the MutS heterodimer. It has been demonstrated that the loss of *Msh4* or *Msh5* results in the earlier loss of prophase I progression, with the almost complete failure of homologous synapsis and cell death prior to pachynema.^2,3^ In a previous study, we identify a homozygous missense mutation (c.1459G>T, p.D487Y) of *MSH5* gene in a pedigree of primary ovarian insufficiency (POI).^4^ In this study, homozygous frameshift truncation and compound heterozygous missense mutations of *MSH5* were identified in three NOA pedigrees.

Three NOA patients (P8944, P7602, and P7824) came from three Chinese families without a history of fertility problems. Known causal factors for male infertility, including cryptorchidism, hypogonadism, cancer, drinking, and smoking, were excluded. Physical examination showed that the development of the penis, epididymis, prostate, scrotum, and vas deferens was normal. The testis size and the FSH level of the three probands were relatively normal. No defect of the 46, XY karyotype or microdeletions on the Y chromosome were detected in these patients (Supplementary Table 1). The results of semen analysis and hematoxylin and eosin (H&E) staining showed that normal sperm were absent in all three probands (Supplementary Table 2 and Supplementary Fig. 1a). TUNEL-positive apoptotic cells were detected in seminiferous tubules (Supplementary Fig. 1b).

To explore the genetic cause of infertility, three probands were chosen for whole-exome sequencing. Frameshift deletion of the *MSH5* gene (c.678_681del, p.Y227Vfs*21) was identified in proband P8944 (Supplementary Table 3). Sanger sequencing (sequencing of blood samples) showed that proband P8944 carried a homozygous frameshift truncation mutation of *MSH5* (c.678_681del). His parents, brother, and sister all carried a heterozygous deletion (Fig. 1a and Supplementary Fig. 2a). The deletion of TTAC causes a frameshift at codon 227 that results in a premature stop codon at codon 247 (Supplementary Fig. 2b). The truncated protein lost 587 amino acids at C-terminal, including the ATP-binding domain. The ATP-binding domain has been demonstrated to be essential for MSH5 function.^2^ We consider this deletion to be equivalent to a null allele, and the inactivation of MSH5 causes defects in germ cell development in the mouse model.^2^ Therefore, we conclude that the four-nucleotide deletion is the pathological cause of NOA in patient P8944.

**Fig. 1:**
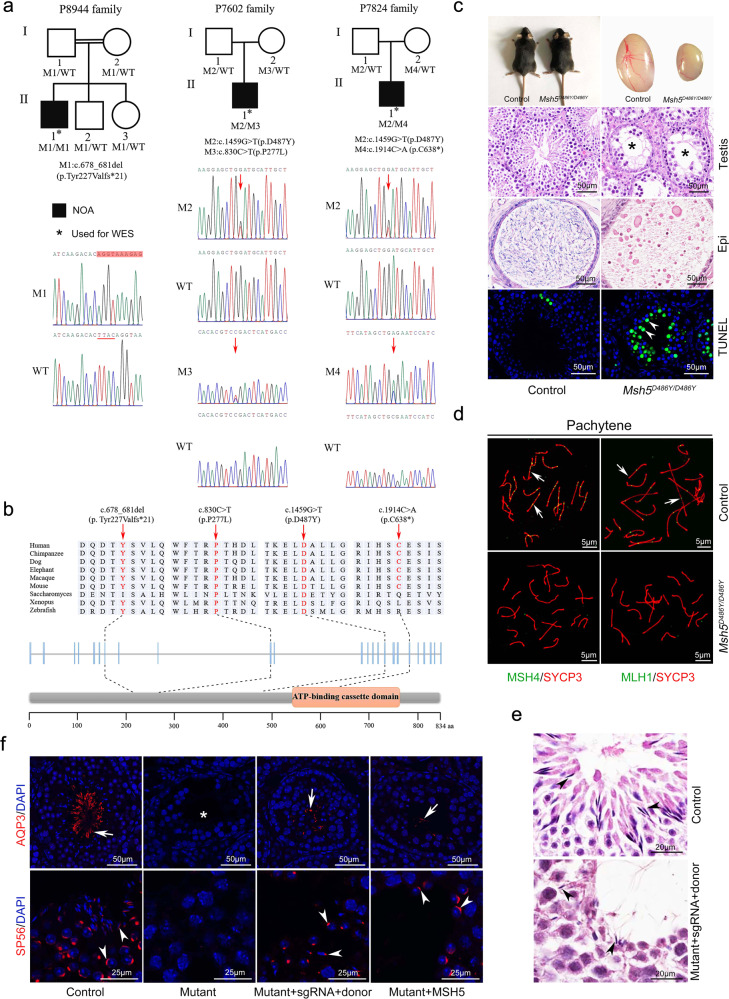
Mutations of *MSH5* in NOA pedigrees and gene editing. **a** The mutations (M1-M4) in *MSH5* were identified in three probands with NOA (P8944, P7602, and P7824). WES was performed in the NOA patients (indicated by asterisks), and the genotypes of other family members were examined by Sanger sequencing. M1 is a frameshift with four nucleotides deletion. M2–M4 are missense point mutations. Red box indicates the shifted sequences, and the deleted nucleotides (TTAC) are indicated with red underline. Red arrows indicate the positions of point mutations. Double horizontal lines represent consanguineous marriages. WT wild-type, M mutant type. **b** The location and conservation of four mutations in MSH5 (M1–M4) protein. **c** The defect of germ cell development in male *Msh5*^*D486Y/D486Y*^ mice. *Msh5*^*D486Y/D486Y*^ male mice were viable at birth and no obvious developmental defects were observed in adults. The size of testes from adult *Msh5*^*D486Y/D486Y*^ mice was significantly reduced compared to that of control littermates. Control testis sections showed normal cell populations within the seminiferous epithelium, whereas germ cell loss no round and elongating spermatids were observed in *Msh5*^*D486Y/D486Y*^ mice (asterisks). A large number of mature sperm were observed in the epididymis cauda of control mice. Only cell debris was observed in the epididymis cauda of *Msh5*^*D486Y/D486Y*^ mice. Very few TUNEL-positive germ cells were observed in control testes and the number of apoptotic cells was significantly increased in *Msh5*^*D486Y/D486Y*^ testes (arrowheads). Scale bars: 50 μm. Experiments were repeated ≥3 times. **d** Immunostaining of MSH4 and MLH1 was performed in chromosome spreads of control and *Msh5*^*D486Y/D486Y*^ germ cells at P30. Synaptonemal complex was labeled with SYCP3 (red). MSH4 and MLH1 foci were observed in control germ cells at the pachytene stage. No MSH4 and MLH1 foci were observed in *Msh5* mutant germ cells at the pachytene stage. Scale bars: 50 μm. Experiments were repeated ≥3 times. **e** H&E staining of control and rescued (with guide RNA, Cas9-expressing vector, and donor DNA) testes. The germ cells at different developmental stages were observed in control testes. A small number of mature sperm were noted in the seminiferous tubules of rescued testes (black arrows). Scale bars: 20 μm. Experiments were repeated ≥3 times. **f** Immunofluorescence analysis of AQP3 and SP56 in control and rescued mutant mice. Mature sperm were also observed in the *Msh5* mutant testes after electroporation with the *Msh5*-expressing vector. The sperm tail was labeled with AQP3 (white arrows). The acrosome of sperm head was labeled with SP56 (white arrowheads). Scale bars: 50 μm, 25 μm. Experiments were repeated ≥3 times

Compound heterozygous mutations of the *MSH5* gene were identified in patients P7824 and P7602 (Supplementary Tables 4 and 5). Both of them carried a common point mutation c.1459G>T (p.D487Y), which we previously identified in the POI pedigree.^4^ In addition to this common mutation, patient P7602 carried another missense mutation, c.830C>T (p.P277L), and patient P7824 carried point mutation at 1914 (c.1914C>A, p.C638*) which causes early termination of translation (Fig. 1a and Supplementary Fig. 2c). The positions of all these mutations are conserved among mammalians (Fig. 1b). Furthermore, the *MSH5* c.1459G>T mutation that has been identified in patients with POI was detected in the fathers of both patients, and female mice carrying the homozygous mutation (p.D486Y) are infertile with atrophic ovaries.^4^

To test the function of this mutation, we examined germ cell development in male mice carrying homozygous *Msh5* mutation. We found that the homozygous *Msh5* mutant (p.D486Y) male mice were infertile and the size of testes was dramatically reduced, and a large number of TUNEL-positive apoptotic cells were noted in the seminiferous tubules of *Msh5*^*D486Y/D486Y*^ mice (Fig. 1c). It is worth noting that the difference of testes size between mice and patients could be due to the different functions of MSH5 in human and mouse. The number of germ cells was significantly reduced in *Msh5*^*D486Y/D486Y*^ mice from P14 (Supplementary Fig. 3). The pathological change observed in *Msh5*^*D486Y/D486Y*^ mice was similar to that in patients P7602 and P7824, indicating that this mutation is a possible etiological cause of NOA.

To explore the causes of the defect of germ cell development in *Msh5*^*D486Y/D486Y*^ mice, the expression of meiosis-associated genes was examined by immunofluorescence. We found that the number of germ cells at the zygotene stage was significantly increased, whereas that at the diplotene and diakinesis stages was significantly decreased in *Msh5*^*D486Y/D486Y*^ mice. And the proportion of unsynapsed homologous chromosomes was increased in *Msh5*^*D486Y/D486Y*^ mice (50.42 ± 1.15%) at the diplotene stage compared to that in control mice (14.14 ± 2.92%) (Supplementary Fig. 4). These results indicate that the formation of the synaptonemal complex (SC) is not affected in *Msh5* mutant germ cells. However, this mutation affects the stability of the SCs, which leads to early desynapsis of homologous chromosomes. Very weak γH2AX signal was retained on autosomes of *Msh5*^*D486Y/D486Y*^ germ cells at the pachytene and diplotene stages, and a small number of RAD51and DMC1 foci were retained in *Msh5* mutant spermatocytes at diplotene (Supplementary Fig. 5), indicating that the DSB is not completely repaired. The point mutation of *MSH5* does not affect the interaction between the MSH4 and MSH5 proteins and the foci of MSH4 and MLH1 was virtually absent in *Msh5* mutant germ cells (Fig. 1d and Supplementary Fig. 6). p.D487Y located in the DNA-binding domain and the original residue is highly conserved among species from yeast to human. We assume that the mutation could affect the combining capacity between MSH4-MSH5 and DNA. These results indicate that the homologous recombination does not occur in *Msh5* mutant germ cells which is consistent with previous studies.

CRISPR/Cas system is the most powerful gene-editing technology which has been widely used in the treatment of different kinds of disease models. To rescue the defect of germ cell development in *Msh5*^*D486Y/D486Y*^ mice, we established an in vivo electroporation system as described previously^5^ (Supplementary Figs. 7a and 8a). The mixture of guide RNA, Cas9-expressing vector, and single-stranded DNA template (donor) or wild-type *Msh5*-expressing vector was injected into the seminiferous tubules. The testes were electroporated after injection to promote the efficiency of transfection. Interestingly, a small number of mature sperm with head and tail were noted in the seminiferous tubules of *Msh5* mutant testes after electroporation 5 weeks later (Fig. 1e, f). However, no mature sperm were obtained from epididymis cauda. This is probably due to the relatively low efficiency of gene correction with this system. The delivery system is needed to be optimized to increase the transfecting efficiency (the experiment is repeated more than three times, and the number of animals is more than ten). Approximately 9% of G>T mutation was corrected in *Msh5* mutant testes electroporated with the guide RNA, Cas9-expressing vector, and donor DNA fragment, and no off-target effects were detected by DNA sequencing (Supplementary Fig. 8 and Supplementary Table 6). These results indicate that the mutation in the germ cells of *Msh5*^*D486Y/D486Y*^ mice could be corrected by in vivo gene editing.

Taken together, our findings based on both human subjects and mouse models strongly suggest that the mutation of *MSH5* is a potential etiological cause of NOA. Most importantly, we have corrected the *Msh5* mutation in the mouse model via in vivo gene editing. This approach will be potentially applied in the clinic treatment of male reproductive diseases.

## Supplementary information

Supplementary Materials

Supplementary Materials Table S3-S5

Informed consent

## Data Availability

The online version of this article contains supplementary material, which is available to authorized users.
