# Supplementary material for: Mutations of MSH5 in nonobstructive azoospermia (NOA) and rescued via in vivo gene editing
Source: Signal Transduct Target Ther. 2022 Jan 3;7:1. doi: 10.1038/s41392-021-00710-4 (PMC8724278; doi:10.1038/s41392-021-00710-4)
Supplement: Supplementary file 1 — Supplementary Materials [file 41392_2021_710_MOESM1_ESM.docx]

Supplementary Materials for

**Mutations of *MSH5* in nonobstructive azoospermia (NOA) and rescued via in vivo gene editing**

Min Chen^1^, Chencheng Yao^2^, Yingying Qin^3^, Xiuhong Cui^4^, Peng Li^2^, Zhiyong Ji^2^, Limei Lin^4,5,6^, Haowei Wu^4,5,6^, Zhi Zhou^7^, Yaoting Gui^1^, Zheng Li^2^ and Fei Gao^4,5,6^

These authors contributed equally: Min Chen, Chencheng Yao, Yingying Qin

Correspondence: Yaoting Gui (guiyaoting2007@aliyun.com) or Zheng Li (lizhengboshi@163.com) or Fei Gao (gaof@ioz.ac.cn)

**This file includes:**

Figure. S1-S8

Table S1, S2 and S6

**Materials and methods**

*Study approval*

This study was approved by the Institutional Ethical Review Committee of Shanghai General Hospital, Shanghai Jiao Tong University (Permit Number 2018KY052), and informed consent for the use of blood and biopsy samples for research was obtained from the donors. In this study, 390 patients with idiopathic NOA (nonobstructive azoospermia) were selected from the cohort study. On the basis of the WHO guidelines (5th edition), at least 3 routine semen analyses were conducted in all the three NOA-affected patients (P8944, P7824 and P7602) and revealed normal semen volumes and complete azoospermia after centrifugation (3000 g, 15 min). Individuals with congenital reproductive diseases such as Klinefelter syndrome or genomic AZF deletions and those in whom other azoospermia-associated factors were recorded, including varicoceles, radiation, chemotherapy, orchitis, cryptorchidism and testicular cancer were excluded from this study.

*Exome sequencing and data analysis*

The blood samples of these patients were subjected to WES (Whole-exome sequencing, Carrier Gene, Shanghai, China). Genomic DNA (gDNA) was extracted from the blood samples of the NOA patients using the TIANamp Blood DNA Kit (TIANGEN) according to the manufacturer’s instructions. DNA was fragmented through Covaris focused ultrasonication. Known exons and exon-intron boundary sequences were captured using the xGen® Exome Research Panel (IDT, USA), and DNA sequencing libraries were prepared following the manufacturer’s instructions. Sequencing was performed on the Illumina HiSeq X10 platform. Sequencing reads were aligned to the human genome (GRCh37/hg19) using Burrows-Wheeler Aligner (BWA). Both single-nucleotide variants (SNVs) and indels within the captured coding exonic intervals were called using GATK, Platypus, VarScan, LoFreq, FreeBayes, SNVer, SAMtools and VarDict. Genetic variants with allele frequencies higher than 1% according to the ExAC Browser and the 1000 Genomes Project were excluded. Additionally, intronic, upstream and downstream variants were removed. Nonsense, frameshift, essential splice-site and potentially deleterious missense (SIFT, PoyPhen-2, and MutationTaster) variants were retained for further analysis. The target fragments of MSH5 were amplified by PCR. The PCR products were purified, labeled with BigDye (Terminatorv3.1 Cycle Sequencing Kits, Applied Biosystems), and sequenced in an ABI 37306l DNA Analyzer (Applied Biosystems, Foster City, CA).

According to the American College of Medical Genetics and Genomics and the Association for Molecular Pathology (ACMG/AMP) guidelines, all the MSH5 mutations were assessed as deleterious.

*Mice*

All animal experimental procedures involved were performed in accordance with protocols approved by the Institutional Animal Care and Use Committee (IACUC) of the Institute of Zoology, CAS (Permit Number AEI-09-07-2014). All mice were maintained in a C57BL/6;129/SvEv mixed background. *Msh5^D486Y/D486Y^* mice were obtained by intercrossing of *Msh5^+/D486Y^* mice. DNA isolated from tail biopsies was used for genotyping by PCR and Sanger sequencing as described previously (Gao et al., 2006; Wang et al., 2013). The primers were as follows: Forward primer, 5’-CCCAAGGGATGAAAAGCCAC-3’; Reverse primer,5’-GATACAGGGAGAGTAATGCGGTCTC-3’.

*Chromosome spread and immunofluorescence*

Testes were decapsulated and incubated with hypotonic extraction buffer (HEB; 30 mM Tris, pH 8.2, 50 mM sucrose, 17 mM trisodium citrate dihydrate, 5 mM EDTA, 0.5 mM DTT, and 0.5 mM PMSF) for 45 min at room temperature. The seminiferous tubules were incubated with 100 μl sucrose (100 mM) after removal of hypotonic extraction buffer. The seminiferous tubules were teared into small pieces with tweezers, and pipetted 10 μl cell suspension onto the slides covered with 500 μl 0.15% Triton X-100 in 1% paraformaldehyde (pH 9.2). Slides were placed in a humid chamber and kept for at least 6 h at room temperature. After air dry, the slides were stored at -80°C for further experiments.

Slides were incubated with 0.4% Kodak Photo-Flo 200 in water for 4 min. After washing with 1🞨 PBST (0.1% Triton X-100 in PBS) for three times, the slides were blocked with 200 μl blocking buffer (3% non-fat milk in 1🞨 PBST) for 1 h at room temperature. After incubating with primary antibodies (3% non-fat milk buffer) overnight at 4°C. Slides were washed with 1🞨 PBST (0.1% Triton X-100 in PBS) for three times and incubated with fluorochrome conjugated secondary antibodies in the dark for 1 h at 37°C. The primary antibodies were diluted as following: SCP3 (1:200, Abcam, ab15093; ab97672), SCP1 (1:200, Abcam, ab15087), γH2AX (1:400, Millipore, 05-636), RAD51 (1:50, Santa Cruz, sc-8349), DMC1 (1:100, Santa Cruz, sc-22768 ), MSH4 (1:100, Abcam, ab58666), MLH1 (1:200, Abcam, ab92312).

*Seminiferous tubule microinjection and In vivo electroporation*

pCS(eGFP) vector was used for CRISPR/Cas9 gene editing. SgRNA guide sequences was cloned into the pCS(eGFP) vector which contain both sgRNA scaffold backbone and Cas9. Completed and sequence-verified vector was was microinjected into cytoplasm of zygotes to examined the cleavage efficiency, and then used for CRISPR/Cas9 gene editing.

The coding sequence of single-guide RNAs (sgRNA) targeting at *Msh5* is “CTATCGTAGCGCCCGGACCAAGG”. The primers of cloning the sgRNA guide sequences into the vector are: F- CACCGCTATCGTAGCGCCCGGACCA; R- AAACTGGTCCGGGCGCTACGATAGC. The sequence of ssDNA donor is “CAGTTTCTCTCAGAGGACAAGCTGCACTATCGTAGCGCCCGGACCAAGGAGCTG**G**ACACGCTGCTGGGAGACCTGCACTGCGAGATCCGGGGTGAGGAGCCCGTGGTAGGAGGGGGCAGGCTGCTCTAAC”, which was synthesized and ULTRAPAGE purified.

The mice were anesthetized and the testes were pulled out from the abdominal cavity. 10 μl of plasmid solution (the mixture of guide RNA, Cas9-expressing vector and ssDNA donor, or wildtype *Msh5*-expressing vector) was injected into the rete testis using with a glass capillary under a stereomicroscope. The testis was squeezed between electrodes, and electroporated four times at 30 V for 50 ms pulse time and 950 ms intervals using an electroporator (BTX, ECM830), then repeated with reverse direction. The histology of testes was examined 5 weeks after electroporation.

*Laser capture microdissection*

5-μm-thick sections were prepared and mounted on untreated glass slides. After deparaffinization, slides were stained with cresyl violet and placed at 40℃ for 20 min. After dissecting with laser-capture microdissection system, the tissue was collected with a special centrifuge tube. 20 μl lysis buffer was added to extract DNA.


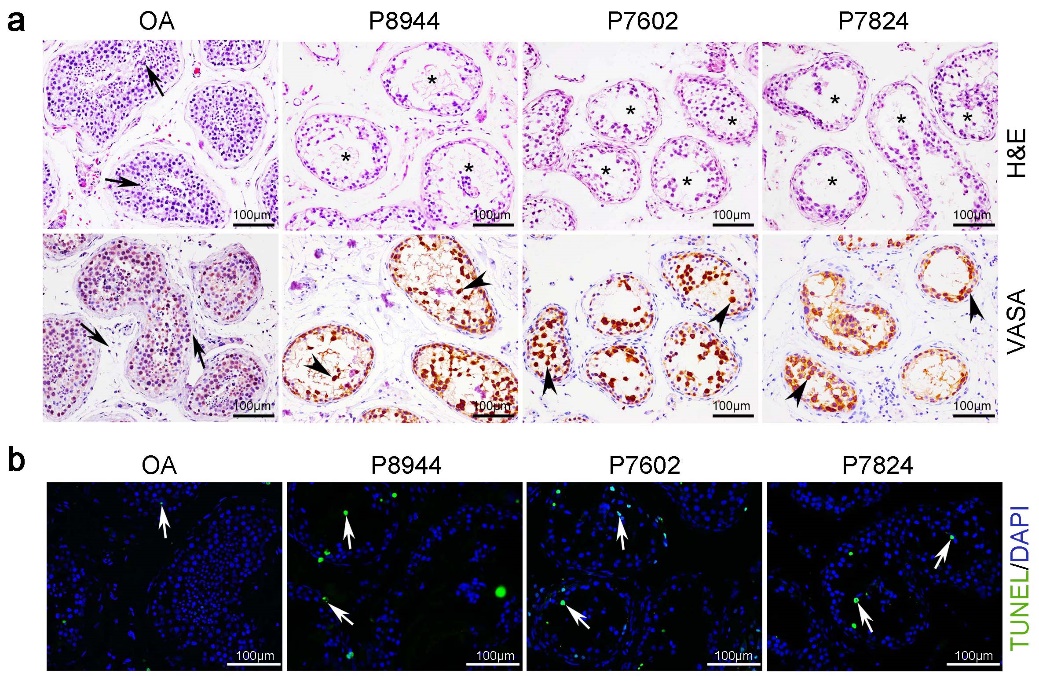


**Figure S1. Histological analysis of the testes of NOA patients.**

(a) The results of Hematoxylin and Eosin (H&E) staining showed the spermatogenesis was disrupted and spermatids were absent in seminiferous tubules of NOA patients (asterisks) compared with OA patients (with normal spermatogenesis). VASA-positive germ cells (arrows) were detected in seminiferous tubules, but all the germ cells were disorganized. Scale bars: 100 μm.

(b) The testicular sections stained for TUNEL (green, arrows). A large number of TUNEL-positive apoptotic cells were noted in the seminiferous tubules of NOA patients. Scale bars: 100 μm.


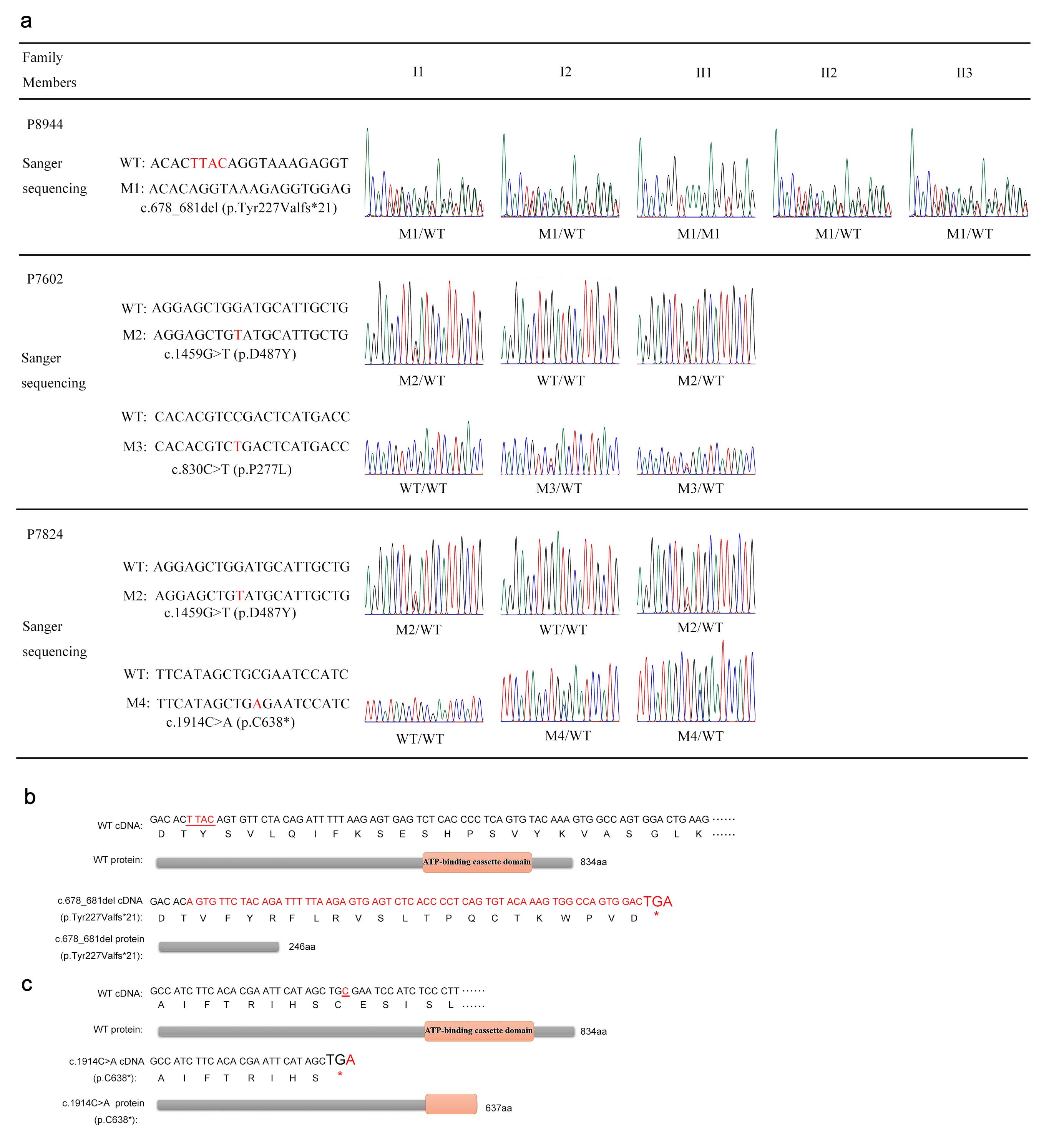


**Figure S2. Sanger sequencing of the MSH5 mutations and schematic of the truncated protein in MSH5 mutations.**

(a) All *MSH5* mutations were verified by Sanger sequencing. The homozygous frameshift mutation in *MSH5* (M1) was identified in P8944. Both of his parents, brother and sister carried a heterozygous deletion. The deleted bases (TTAC) are labeled with red in WT sequence. The compound heterozygous mutations in *MSH5* (M2-M4) were identified in P7602 and P7824, and both of them parents carried the heterozygous mutation respectively. The mutation bases are labeled with red in mutation sequence. WT, wide-type; M, mutant type.

(b) The frameshift truncation mutation (c.678_681del, p.Y227Vfs*21) in *MSH5* cause a frameshift at codon 227 that predicts a truncated protein of 246 amino acids, while the wild type protein consists with 834 amino acids. The deleted bases (TTAC) are labeled with red in WT sequence. The shifted sequences after deletion start points are labeled with red. The termination code (TGA *) is shown in the mutative cDNA.

(c) The point mutation (c.1914C>A, p.C638*) in *MSH5* cause premature translational termination of *MSH5* that predicts a truncated protein of 637 amino acids that result in the loss of the highly conserved amino acids of the full-length protein. The mutation base is labeled with red. The termination code (TGA *) is shown in the mutative cDNA.


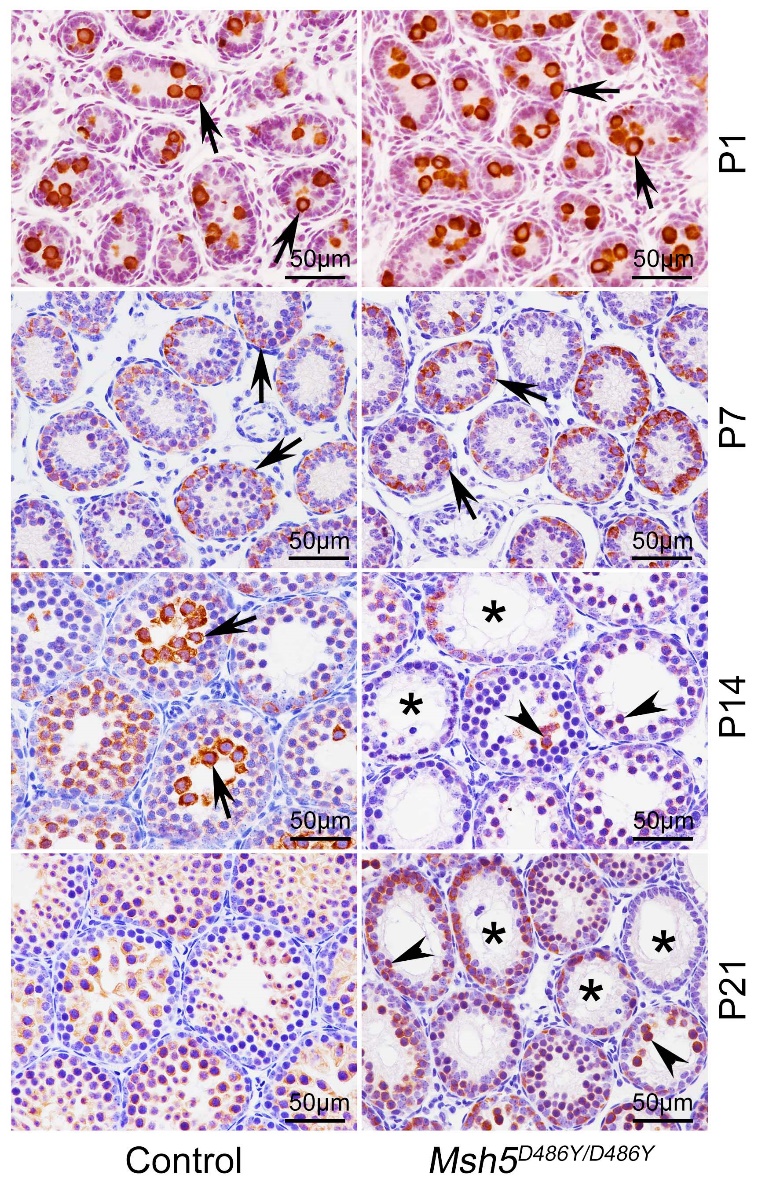


**Figure S3. Germ cell loss is observed in *Msh5^D486Y/D486Y^* testes from P14 onwards.**

Germ cells in control and *Msh5^D486Y/D486Y^* testes were labeled with MVH (brown). No defect of germ cell development was noted in *Msh5^D486Y/D486Y^* testes at P1 (arrows) and P7 (arrows). The number of germ cells was significantly reduced in *Msh5^D486Y/D486Y^* testes at P14 (asterisks) and P21 (asterisks) compared to control testes. Scale bars: 50 μm. Experiments were repeated ≥ 3 times.


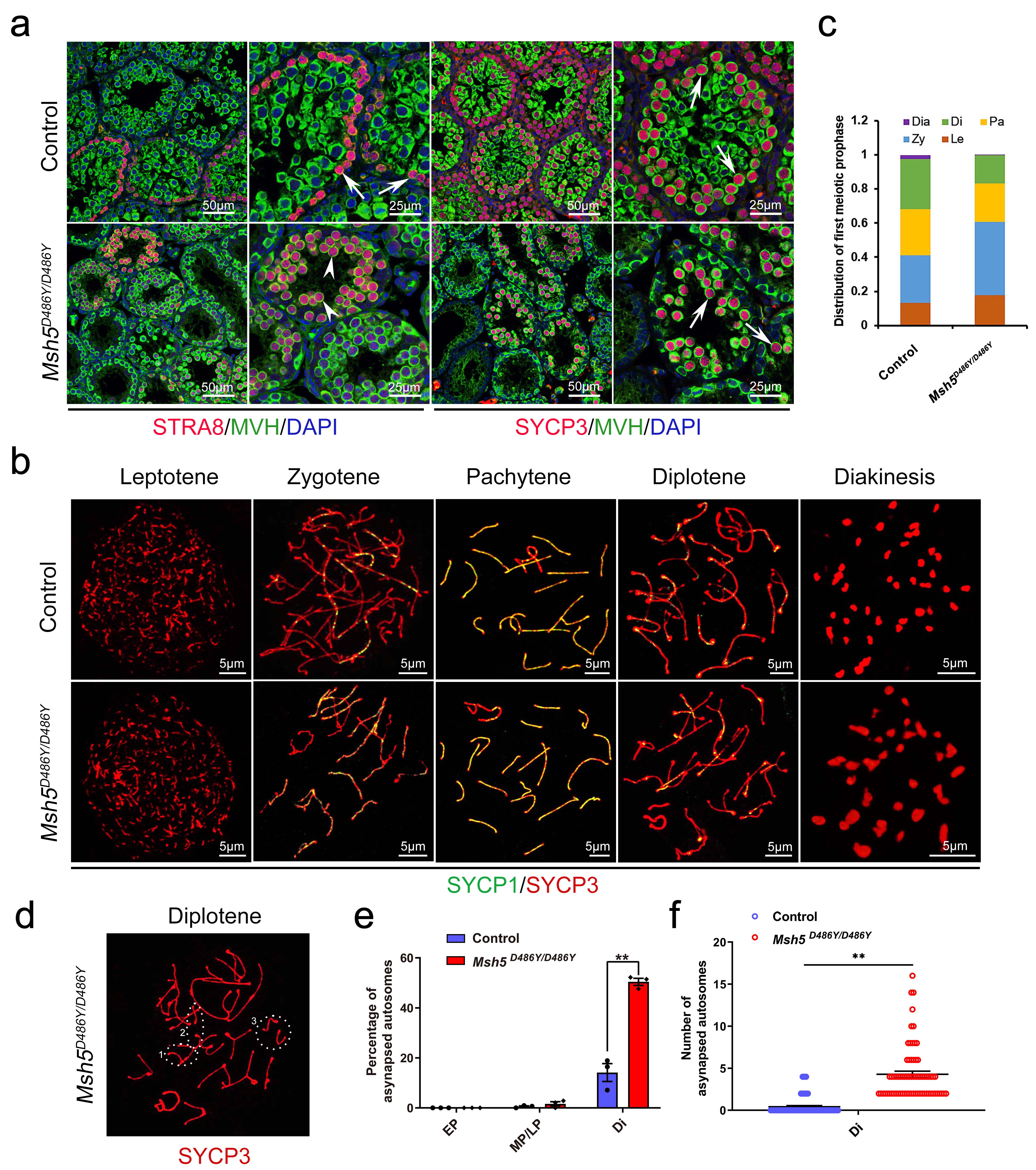


**Figure S4. Aberrant meiosis is observed in *Msh5^D486Y/D486Y^* mice germ cells.**

(a) The expression of STRA8 (red) and SYCP3 (red) was analyzed by immunofluorescence in control and *Msh5^D486Y/D486Y^* testes at P21. Germ cells were labeled with antibody against MVH (green). STRA8-positive germ cells were detected in both control (arrows) and *Msh5^D486Y/D486Y^* testes (arrowheads). SYCP3-positive germ cells were detected in both control (arrows) and *Msh5^D486Y/D486Y^* testes (arrows). Scale bars: 50 μm, 25 μm.

(b) Immunostaining of SYCP1 (green) and SYCP3 (red) was performed in chromosome spreads of spermatocytes from control and *Msh5^D486Y/D486Y^* mice at P30. Scale bars: 5 μm.

(c) Quantitative analysis of germ cells at different stages of prophase Ⅰ in control and *Msh5^D486Y/D486Y^* testes at P30. The number of germ cells at the zygotene stage (Control n = 732/2648; mutant n = 663/1597; P = 1.77E-3) was increased, whereas that at the diplotene (Control n = 794/2648; mutant n = 271/1597; P = 0.022) and diakinesis (Control n = 61/2648; mutant n = 2/1597; P = 3.55E-3) stages was decreased in *Msh5^D486Y/D486Y^* mice.

(d-f) Quantitative analysis of asynapsed autosomes in control and *Msh5^D486Y/D486Y^* spermatocytes. The numbers of asynapsed autosomes per nucleus in *Msh5^D486Y/D486Y^* diplotene spermatocytes (4.28 ± 0.37) was significantly increased compared with control diplotene spermatocytes (0.42 ± 0.13) . Each symbol represents a single nucleus (f). Dotted lines and 1-3 denote the asynapsed autosomes. Le (leptotene), Zy (zygotene), Pa (pachytene), Di (diplotene), Dia (diakinesis), EP (early pachytene), MP/LP (mid pachytene/late pachytene). The data are presented as the mean ± SEM. *, P < 0.05; **, P < 0.01.

Experiments were repeated ≥ 3 times.


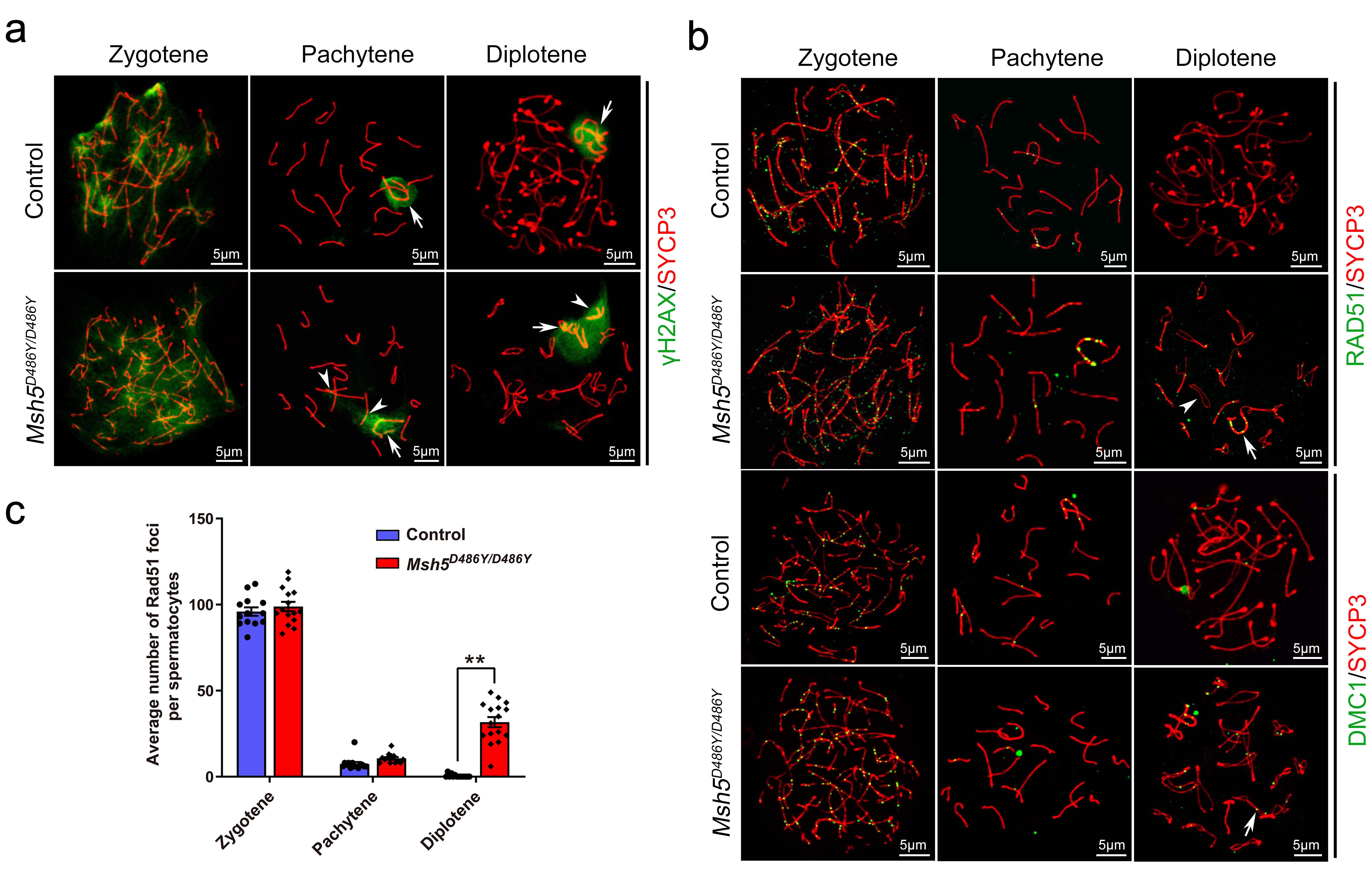


**Figure S5. DSB repair is affected in *Msh5* mutant germ cells.**

Immunostaining of SYCP3 (red), γH2AX (green), RAD51 and DMC1 (green) was performed in chromosome spreads of control and *Msh5^D486Y/D486Y^* mice at P30. (a) Scattered γH2AX signals were detected in both control and *Msh5* mutant germ cells at zygotene stage. γH2AX signals were only detected in the sex body of control germ cells at pachytene (white arrows) and diplotene (white arrows) stages. In addition to the sex body, weak signal of γH2AX signals were observed on autosomal chromosomes of *Msh5* mutant germ cells at pachytene (white arrowheads) and diplotene (white arrowheads) stages. (b) A large number of RAD51 foci were detected in control and *Msh5* mutant germ cells at zygotene stage. The number of RAD51 foci was significantly reduced in both control and *Msh5* mutant germ cells at pachytene and diplotene stages. The results of quantitative analysis showed that the number of RAD51 foci in *Msh5^D486Y/D486Y^* germ cells was significantly increased compared to that in control germ cells at diplotene stage (c). (b) A large number of DMC1 foci were detected in control and *Msh5* mutant germ cells at zygotene stage. The number of DMC1 foci was significantly reduced in both control and *Msh5* mutant germ cells at pachytene and control diplotene stages, while the number of DMC1 foci was increased in *Msh5^D486Y/D486Y^* spermatocytes at diplotene stage. The data are presented as the mean ± SEM. **, P < 0.01. Scale bars: 5 μm. Experiments were repeated ≥ 3 times.


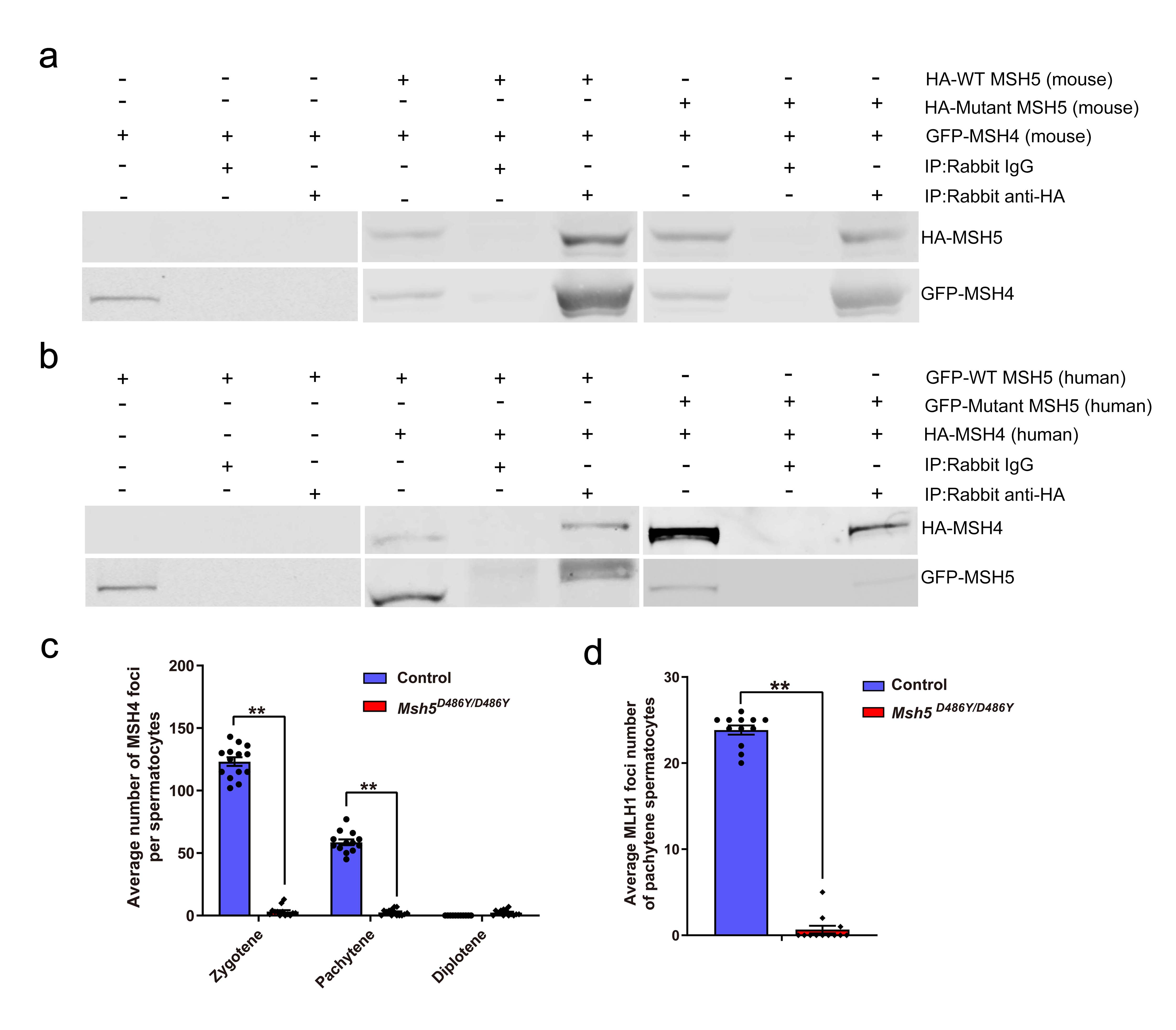


**Figure S6. Point mutation in *MSH5* gene does not affect the interaction between MSH4 and MSH5.**

(a) Mouse MSH4-GFP expression vector in combination with HA-tagged control or mutant mouse MSH5 vector was co-transfected into 293T cells. 293T cells transfected with only MSH4-GFP was used as a negative control.

(b) HA-tagged human MSH4 expression vector in combination with human control or mutant MSH5-GFP vector was co-transfected into 293T cells. 293T cells transfected with only MSH5-GFP was used as a negative control.

Extracted proteins were immunoprecipitated (IP) by anti-HA antibody and then separated by 8% SDS-PAGE. The transferred membrane was immunoblotted with either anti-HA or anti-GFP antibody.

(c-d) Quantitative analysis of MSH4 and MLH1 foci in control and *Msh5^D486Y/D486Y^* germ cells at P30. The data are presented as the mean ± SEM. **, P < 0.01.


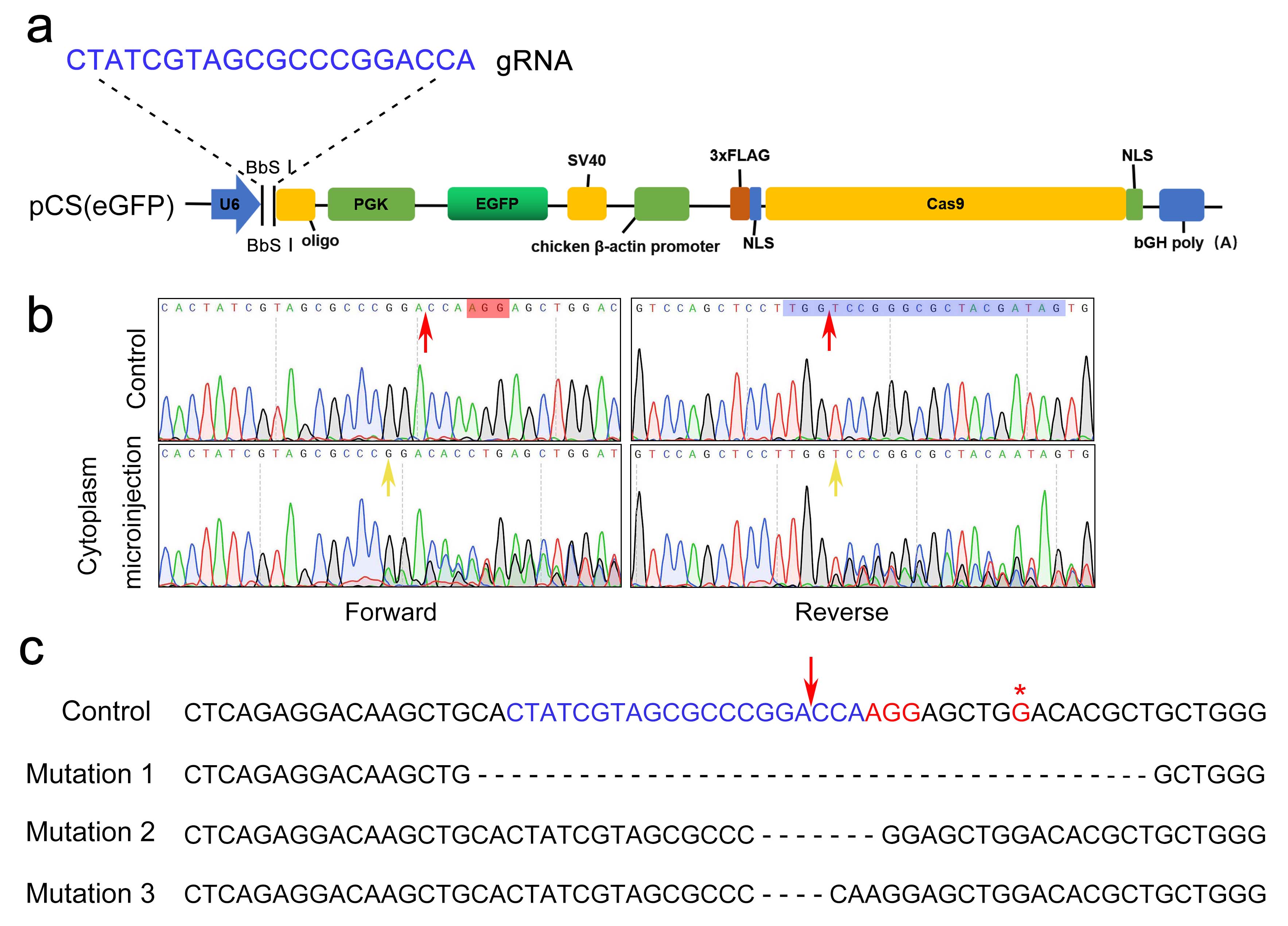


**Figure S7. The construction of the gRNA and Cas9 expression plasmid and examination of cleavage efficiency.**

(a) Schematic diagram for construction of the gRNA and Cas9 expression plasmid pCS(eGFP). The coding sequence of gRNA is labeled in blue.

(b) The pCS(eGFP)-gRNA vector was microinjected into cytoplasm of zygotes, *Msh5* gene sequence was examined by Sanger sequencing. Red box is indicated PAM sequence. Blue box is indicated gRNA target sequence. The cleavage sites of Cas9 are indicated by red arrows. Yellow arrows indicate double peaks.

(c) Cleavage efficiency was examined by TA clone. Individual clones were assayed by PCR and Sanger sequencing (using the M13F primers). Red “G” labeled with asterisk was denoted the mutant base. The cleavage site of Cas9 is indicated by red arrows.


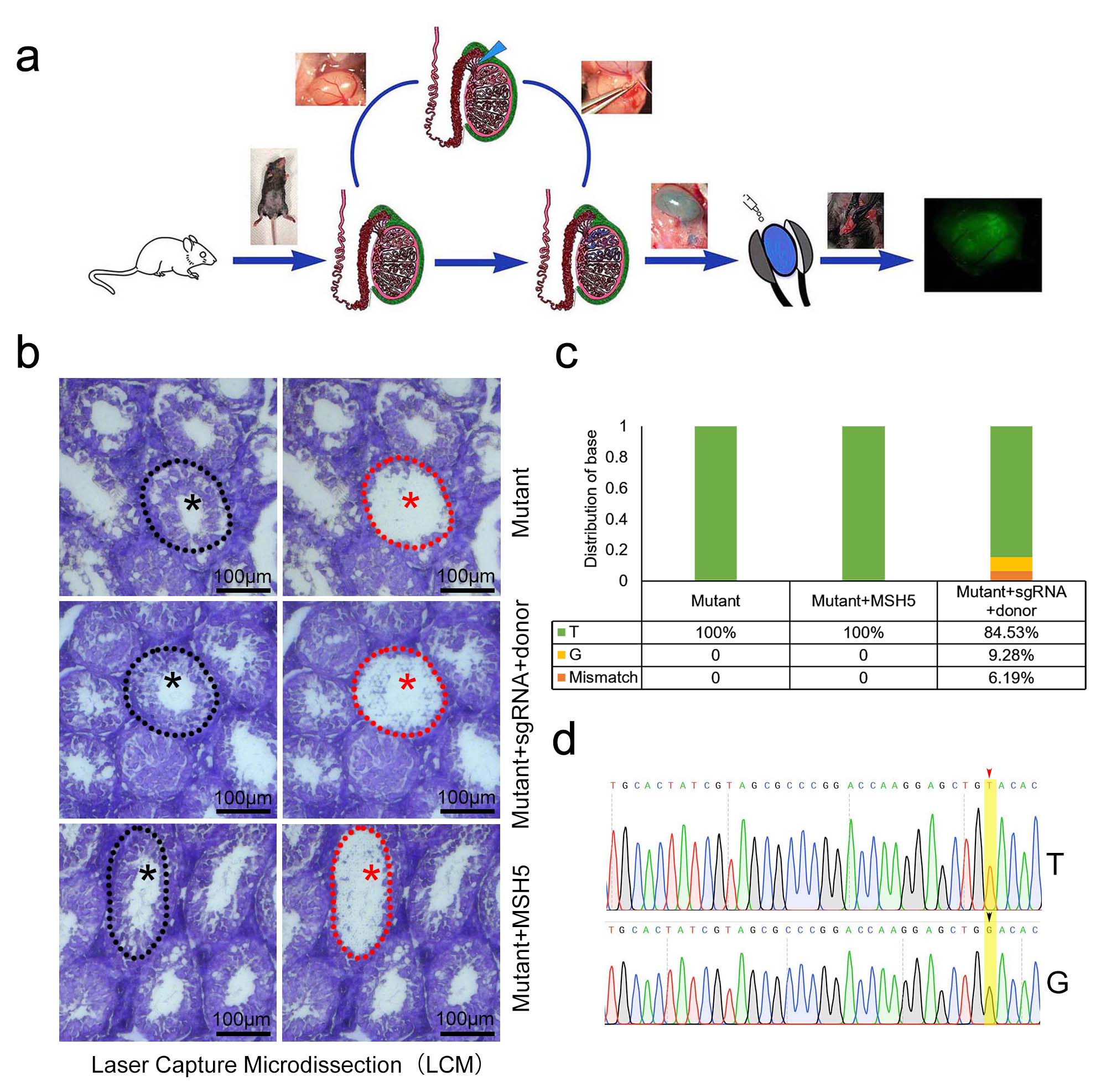


**Figure S8.** **The mutation in the *Msh5* gene is corrected by in vivo gene editing.**

(a) Schematic diagram of in vivo electroporation.

(b) The germ cells from mutant and rescued mice (with the guide RNA, Cas9 and donor or wildtype MSH5) were obtained by laser capture. The black dotted area and asterisk indicate seminiferous tubules before laser capture. The red dotted area and asterisk indicate seminiferous tubules after laser capture. Scale bars: 100 μm.

(c) Quantitative analysis of Sanger sequencing results for mutant and rescued mice.

(d) Sanger sequencing results of the rescued mice (with the guide RNA, Cas9 and donor). Black arrowheads point to the wildtype base G. Red arrowheads point to the mutant base T.

Experiments were repeated ≥ 3 times.

**Table S1. Clinical characteristics of NOA patients with mutations in *MSH5***

| Patient NO. | Mutation | Sequence variation | Amino acid variation | Left  testicular  volume (mL) | Right testicular  volume (mL) | FSH  (IU/L) | LH  (IU/L) | T  (ug/L) | E2  (pg/mL) | PRL  (ng/mL) | Karyotype | YqAZF |
| --- | --- | --- | --- | --- | --- | --- | --- | --- | --- | --- | --- | --- |
| P8944 | Frameshift | c.678_681del | p.(Y227Vfs*21) | 12 | 12 | 10.31 | 5.24 | 1.03 | 9.8 | 13.45 | 46, XY | Yes |
| P7602 | Missense | c.830  C>T | p.(P277L) | 15 | 15 | 3.82 | 2.74 | 3.95 | 11.11 | 10.68 | 46, XY | Yes |
|  | Missense | c.1459  G>T | p.(D487Y) |  |  |  |  |  |  |  |  |  |
| P7824 | Stop gained | c.1914  C>A | p.(C638*) | 12 | 12 | 9.54 | 6.99 | 3.77 | 28.35 | 11.96 | 46, XY | Yes |
|  | Missense | c.1459  G>T | p.(D487Y) |  |  |  |  |  |  |  |  |  |
| Reference Values ^a^ |  |  |  | 12-15 | 12-15 | 1.27-  19.26 | 1.24-  8.62 | 1.75-7.81 | <38.95 | 2.64-  13.13 |  |  |

FSH, follicle-stimulating hormone; LH, luteinizing hormone; T, testosterone; E2, estradiol; PRL, prolactin; YqAZF, azoospermia factor.

^a^ Reference values were suggested by local clinical laboratory.

**Table S2: Semen analysis of the** **NOA-affected patients**

|  | P8944 | P7824 | P7602 | Reference |
| --- | --- | --- | --- | --- |
| Semen volume (mL) | 2.9 ± 0.29 | 3.2 ± 0.15 | 2.5 ± 0.29 | ≥1.5 |
| Concentration (millions/mL) | 0 | 0 | 0 | ≥15 |
| PR (%) | 0 | 0 | 0 | ≥32 |
| NP (%) | 0 | 0 | 0 |  |
| IM (%) | 0 | 0 | 0 |  |
| Centrifuged spermatozoa number (/ejaculate) | 0 | 0 | 0 |  |

Abbreviations: PR, progressive; NP, non-progressive; IM, immotility.

The data are presented as the mean ± SEM.

**Table S6. No off-target is detected in the *Msh5* gRNA**

| Name | Locus | Sequence | Mismatch | Indel |
| --- | --- | --- | --- | --- |
| Target |  | CAAGGAGCTGTACACGCTGCTGG |  |  |
| Off target 1 | chr1：-160097217 | CAAaGAGCgGTACAaGCTGtCGG | 4MMs [4:9:15:20] | ND |
| Off target 2 | chr2：+26591682 | agAGGAGgTGTACAgGCTGCAGG | 4MMs [1:2:8:15] | ND |
| Off target 3 | chr4：-139455494 | CAAtGAGCTtTACAacCTGCTGG | 4MMs [4:10:15:16] | ND |
| Off target 4 | chr5：-24399957 | CccGGAGCTGTACACGgaGCTGG | 4MMs [2:3:17:18] | ND |
| Off target 5 | chr7：-79709164 | CAtGGAGCaGTACAaaCTGCAGG | 4MMs [3:9:15:16] | ND |
| Off target 6 | chr9：-116110006 | CAtcGAGCTGgACACGCTGgTGG | 4MMs [3:4:11:20] | ND |
| Off target 7 | chr11：+119981406 | CAAtGAGCTGctCACGCaGCTGG | 4MMs[4:11:12:18] | ND |
| Off target 8 | chr12：+107915477 | CAgGGtGCTGTAgACGCTGaAGG | 4MMs [3:6:13:20] | ND |
| Off target 9 | chr14：+108911420 | CAcGGAGaTGgACACcCTGCTGG | 4MMs [3:8:11:16] | ND |
| Off target 10 | chr16：+8484835 | CAAGGAGCTGgACtgGCTGaAGG | 4MMs[11:14:15:20] | ND |

10 potential off-targeting locus were predicted by bioinformatic analysis. After sequencing analysis, no mutation or deletion was detected at these loci.
