# Supplementary material for: Mutations of MSH5 in nonobstructive azoospermia (NOA) and rescued via in vivo gene editing
Source: Signal Transduct Target Ther. 2022 Jan 3;7:1. doi: 10.1038/s41392-021-00710-4 (PMC8724278; doi:10.1038/s41392-021-00710-4)
Supplement: Supplementary file 3 — Informed consent [file 41392_2021_710_MOESM3_ESM.pdf]

上海市第一人民醫院
SHANGHAI GENERAL HOSPITAL
上海交通大學附属第一人民医院
上 海 市 红 十 字 医 院

无精子症手术剩余组织用于科学研究的知情同意书

我______________因患 无精子症 授权上海交通大学附属第一人民医院泌尿外科临床医学中心男科为我实施睾丸取精术或输精管道探查（吻合）手术。

第一人民医院医生已向我详细介绍有关睾丸取精术与精道探查手术的适应症。我已经了解，无精子症分为梗阻性与非梗阻性。非梗阻性无精子症为睾丸生精功能衰竭所致，目前尚无较好的药物治疗方法，但睾丸仍有局灶性的生精部位，或个别生精小管有精子发生。出于个人情感及伦理学考虑，自愿将睾丸取精手术的睾丸组织结合精子冷冻、单精子卵胞浆注射（ICSI）作为第一治疗选择。国外与国内的研究报道均证实，部分非梗阻性无精子症患者可通过手术获取精子，结合 ICSI 技术，生育正常后代。在慎重考虑本人疾病现状及可能产生的不利治疗后果，我自愿接受睾丸取精手术。医生已经向我介绍了第一人民医院所采用的睾丸取精术，手术在全麻或腰麻下实施，手术操作在显微镜下进行，尽可能避免睾丸供养血管的损伤。显微镜下有目的地选取管腔较为粗大的生精小管，既可提高精子的获取率，又减少组织的损伤量，术后血肿及雄激素水平下降的发生率较前降低。如取得的睾丸组织在手术当时未检出精子，还可采用特定培养方法在体外继续培养，如培养成功，可将精子冻存备辅助生殖治疗用。该治疗方法的常见并发症为：伤口感染；伤口渗血；阴囊血肿形成；一过性雄激素水平下降。

如果证实为梗阻性无精子症，医师将为我实施精道探查手术，根据病情可能实施附睾输精管的吻合、输精管与输精管吻合、射精管切开手术等等。我已经完全了解，该手术目前大约有 60%-70%的手术成功率，对手术失败的风险我已经完全知晓。我理解一旦手术失败，我可以再选择 ICSI 助孕生育子代。

我确认：我已经充分了解以上手术相关内容。第一人民医院医生将会为我的生育成功做最大的努力，在保证我生育能力的前提下，为了生殖医学事业的发展，术中睾丸组织镜检为医学常规，我志愿将其检查后剩余的睾丸组织捐献用于科学研究。这些捐赠是我本人的意愿，完全自愿，没有人强迫我参加这项研究；我捐赠这些组织或血样决非为了任何金钱报酬或换取其他利益；有关人员已经将这项研究的目的和过程向我解释清楚；我所捐献的组织在实验结束的时候都会按照正规途径销毁；有关人员已经将这项研究的潜在价值向我解释清楚。我同意不参与分享这次捐献可能带来的研究成果；我的这一捐献行为及我的个人资料将得到严格保密，在任何情况下都不会泄露；我可以随时退出此项研究，而且不会因为退出而受到任何处罚或有任何利益上的损失。我已认真阅读本知情同意书，有关问题已经与医生进行了相关讨论，并得到了满意答复。

我自愿选择本手术，并签署本知情同意书。

患者签字：[illegible]　　　　手术者签字：
时　间：2019、2.11　　　　时　间：

无精子症手术剩余组织用于科学研究的知情同意书

我[illegible]因患无精子症授权上海交通大学附属第一人民医院泌尿外科临床医学中心男科为我实施睾丸取精术或输精管道探查（吻合）手术。

第一人民医院医生已向我详细介绍有关睾丸取精术与精道探查手术的适应症。我已经了解，无精子症分为梗阻性与非梗阻性。非梗阻性无精子症为睾丸生精功能衰竭所致，目前尚无较好的药物治疗方法，但睾丸仍有局灶性的生精部位，或个别生精小管有精子发生。出于个人情感及伦理学考虑，自愿将睾丸取精手术的睾丸组织结合精子冷冻、单精子卵胞浆注射（ICSI）作为第一治疗选择。国外与国内的研究报道均证实，部分非梗阻性无精子症患者可通过手术获取精子，结合 ICSI 技术，生育正常后代。在慎重考虑本人疾病现状及可能产生的不利治疗后果，我自愿接受睾丸取精手术。医生已经向我介绍了第一人民医院所采用的睾丸取精术，手术在全麻或腰麻下实施，手术操作在显微镜下进行，尽可能避免睾丸供养血管的损伤。显微镜下有目的地选取管腔较为粗大的生精小管，既可提高精子的获取率，又减少组织的损伤量，术后血肿及雄激素水平下降的发生率较前降低。如取得的睾丸组织在手术当时未检出精子，还可采用特定培养方法在体外继续培养，如培养成功，可将精子冻存备辅助生殖治疗用。该治疗方法的常见并发症为：伤口感染；伤口渗血；阴囊血肿形成；一过性雄激素水平下降。

如果证实为梗阻性无精子症，医师将为我实施精道探查手术，根据病情可能实施附睾输精管的吻合、输精管与输精管吻合、射精管切开手术等等。我已经完全了解，该手术目前大约有 60%-70%的手术成功率，对手术失败的风险我已经完全知晓。我理解一旦手术失败，我可以再选择 ICSI 助孕生育子代。

我确认：我已经充分了解以上手术相关内容。第一人民医院医生将会为我的生育成功做最大的努力，在保证我生育能力的前提下，为了生殖医学事业的发展，术中睾丸组织镜检为医学常规，我志愿将其检查后剩余的睾丸组织捐献用于科学研究。这些捐赠是我本人的意愿，完全自愿，没有人强迫我参加这项研究；我捐赠这些组织或血样决非为了任何金钱报酬或换取其他利益；有关人员已经将这项研究的目的和过程向我解释清楚；我所捐献的组织在实验结束的时候都会按照正规途径销毁；有关人员已经将这项研究的潜在价值向我解释清楚。我同意不参与分享这次捐献可能带来的研究成果；我的这一捐献行为及我的个人资料将得到严格保密，在任何情况下都不会泄露；我可以随时退出此项研究，而且不会因为退出而受到任何处罚或有任何利益上的损失。我已认真阅读本知情同意书，有关问题已经与医生进行了相关讨论，并得到了满意答复。

我自愿选择本手术，并签署本知情同意书。

患者签字：[illegible]　　　手术者签字：

时　　间：2018.8.7　　　时　　间：

无精子症手术剩余组织用于科学研究的知情同意书

我____________因患 无精子症 授权上海交通大学附属第一人民医院泌尿外科临床医学中心男科为我实施睾丸取精术或输精管道探查（吻合）手术。

第一人民医院医生已向我详细介绍有关睾丸取精术与精道探查手术的适应症。我已经了解，无精子症分为梗阻性与非梗阻性。非梗阻性无精子症为睾丸生精功能衰竭所致，目前尚无较好的药物治疗方法，但睾丸仍有局灶性的生精部位，或个别生精小管有精子发生。出于个人情感及伦理学考虑，自愿将睾丸取精手术的睾丸组织结合精子冷冻、单精子卵胞浆注射（ICSI）作为第一治疗选择。国外与国内的研究报道均证实，部分非梗阻性无精子症患者可通过手术获取精子，结合 ICSI 技术，生育正常后代。在慎重考虑本人疾病现状及可能产生的不利治疗后果，我自愿接受睾丸取精手术。医生已经向我介绍了第一人民医院所采用的睾丸取精术，手术在全麻或腰麻下实施，手术操作在显微镜下进行，尽可能避免睾丸供养血管的损伤。显微镜下有目的地选取管腔较为粗大的生精小管，既可提高精子的获取率，又减少组织的损伤量，术后血肿及雄激素水平下降的发生率较前降低。如取得的睾丸组织在手术当时未检出精子，还可采用特定培养方法在体外继续培养，如培养成功，可将精子冻存备辅助生殖治疗用。该治疗方法的常见并发症为：伤口感染；伤口渗血；阴囊血肿形成；一过性雄激素水平下降。

如果证实为梗阻性无精子症，医师将为我实施精道探查手术，根据病情可能实施附睾输精管的吻合、输精管与输精管吻合、射精管切开手术等等。我已经完全了解，该手术目前大约有 60%-70%的手术成功率，对手术失败的风险我已经完全知晓。我理解一旦手术失败，我可以再选择 ICSI 助孕生育子代。

我确认：我已经充分了解以上手术相关内容。第一人民医院医生将会为我的生育成功做最大的努力，在保证我生育能力的前提下，为了生殖医学事业的发展，术中睾丸组织镜检为医学常规，我志愿将其检查后剩余的睾丸组织捐献用于科学研究。这些捐赠是我本人的意愿，完全自愿，没有人强迫我参加这项研究；我捐赠这些组织或血样决非为了任何金钱报酬或换取其他利益；有关人员已经将这项研究的目的和过程向我解释清楚；我所捐献的组织在实验结束的时候都会按照正规途径销毁；有关人员已经将这项研究的潜在价值向我解释清楚。我同意不参与分享这次捐献可能带来的研究成果；我的这一捐献行为及我的个人资料将得到严格保密，在任何情况下都不会泄露；我可以随时退出此项研究，而且不会因为退出而受到任何处罚或有任何利益上的损失。我已认真阅读本知情同意书，有关问题已经与医生进行了相关讨论，并得到了满意答复。

我自愿选择本手术，并签署本知情同意书。

患者签字：盛新　　　　手术者签字：[illegible]

时　间：2018.6.4　　　　时　间：2018.6.21
